# Exploring the relationship between bone density and severity of distal radius fragility fracture in women

**DOI:** 10.1186/s13018-014-0057-8

**Published:** 2014-07-17

**Authors:** Alvilde Dhainaut, Kamil Daibes, Adalsteinn Odinsson, Mari Hoff, Unni Syversen, Glenn Haugeberg

**Affiliations:** INM Norwegian University of Science and Technology, Trondheim, 7491 Norway; Department of Orthopedic Surgery, Sørlandet Hospital, Kristiansand, S 4604 Norway; Department of Orthopaedic Surgery, St. Olav’s Hospital, Trondheim, 7030 Norway; Department of Rheumatology, St. Olav’s Hospital, Trondheim, 7030 Norway; Institute of Cancer Research and Molecular Medicine, NTNU, Trondheim, 7491 Norway; Department of Endocrinology, St. Olav’s Hospital, Trondheim, 7030 Norway; Department of Rheumatology, Sørlandet Hospital, Kristiansand, S 4604 Norway; MTFS-Department of Neuroscience, Division of Rheumatology, University Hospital of Trondheim, Norwegian University of Science and Technology, Trondheim, 7489 Norway

**Keywords:** Bone mineral density, Digital X-ray radiogrammetry, Dual energy X-ray absorptiometry, Distal radius, Fracture, Severity

## Abstract

**Background:**

Bone mineral density (BMD) has been shown to be a consistent and independent risk factor for distal radius fracture. Inconsistent data have been reported on the association between BMD and severity of distal radius fracture. Our primary aim was to explore if there is an association between cortical BMD at the hand and the severity of fragility distal radius fracture.

**Methods:**

Consecutively recruited females aged ≥50 years with fragility fracture at the distal radius (*n* = 110) from a county hospital were included. Cortical hand BMD was assessed by the digital X-ray radiogrammetry (DXR) method. X-rays of the fracture were scored by experienced orthopedic surgeons for fracture severity according to the Müller AO classification of long bones and radiographic parameters such as ulnar variance and dorsal angle.

**Results:**

A weak association between lower DXR BMD and increased ulnar variance and dorsal angle was found but not with the AO scoring system for fracture type. A history of glucocorticoid (GC) use but not DXR-BMD was found to be significantly associated with the presence of having an intra- or extra-articular fracture.

**Conclusion:**

Our data indicate that bone material properties which are impaired by GC use are more important for fracture severity than BMD.

## Introduction

Distal radius fracture is one of the most common osteoporotic fractures in the elderly [1]. Reduced bone mineral density (BMD) has been identified as one of the most significant risk factors for distal radius fracture [2-4]. Patients with distal radius fracture are either treated conservatively with a plaster cast or by surgery depending on the severity and the instability of the fracture [5]. In a human cadaver study, reduced forearm BMD was found to correlate to the severity of the distal radius fracture [6]. The results from *in vivo* studies exploring the possible association between dual energy X-ray assessed (DXA)-BMD and the severity of distal radius fracture have been inconsistent [7-11]. During the last years, there has been an increased interest illuminating the relationship between cortical bone and fractures [11,12]. In the study by Xie and Barenholdt, a significant lower cortical bone density assessed with peripheral quantitative computer tomography (pQCT) was found in individuals with displaced, compared to individuals with undisplaced fractures, whereas no significant difference was found between the two patient groups for DXA-BMD [11]. Digital X-ray radiogrammetry (DXR) is a feasible method which calculates cortical BMD in the metacarpal bones from plain X-rays [13,14]. Assessment by this method has revealed a significant reduction in DXR-BMD in patients with distal radius fracture compared with individuals in the general population and has been shown to be a predictor and risk factor for distal radius fracture and hip fracture [15-17]. The primary aim of the present study was to explore if there also is an association between reduced cortical hand BMD measured with DXR and fracture severity.

## Material and methods

### Subjects

The study population consisted of women aged 50 years or more with a recent fragility fracture in distal radius consecutively recruited from a regional hospital during 2004 and 2005. A total of 278 women were identified; among them, 218 were assessed at the osteoporosis center where demographic, clinical and treatment data were collected, and DXA at the spine (L2-4), total hip, and femoral neck was performed. Among the 218 women, 110 had both radiographs for DXR-BMD calculation and for AO scoring performed at the fracture assessment point.

### Bone density measures

The hand DXR-BMD was calculated using the Sectra DXR software (Sectra, Linköping, Sweden). DXR calculates a mean BMD from metric measures of cortical thickness at the second, third, and fourth metacarpal bones on standardized hand X-rays. The formula includes an estimated porosity aimed to be the fraction of cortical bone not occupied by bone, as described previously [13]. The hand X-rays for DXR-BMD calculation were acquired with Fuji FCR XG1 (CR; FFD 100 cm; tube voltage 50 kV; exposure dose 5 mA), and the non-dominant hand was used when possible (93/110).

DXA-BMD at the femoral neck, total hip, and spine (L2–4) was assessed using Lunar Prodigy with enCORE software (GE Healthcare, Madison, WI, USA).

Trained nurses performed all the BMD measurements using standardized protocols. The *in vivo* coefficient of variation (CV%) for DXR at the non-dominant hand was 0.46% and for DXA at the femoral neck 1.68%, total hip 0.88%, and spine (L2–4) 1.26%.

### X-ray and clinical fracture severity

Radiographic fracture severity was assessed and scored on the routine standard radiographs (AP and lateral view) used to diagnose the fracture at first visit after trauma. The radiographs were analyzed by trained orthopedic physicians (KD and AO) using the digital hospital X-ray system PACS.

**Figure 1:**
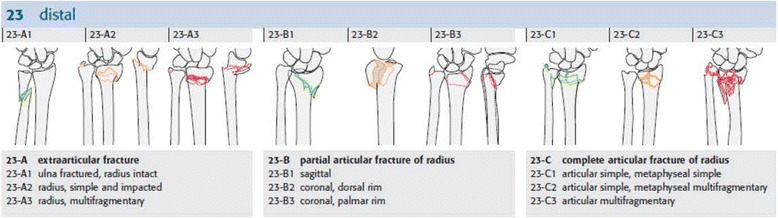
**The Müller AO Classification of distal radius fracture.** Copyright by AO Foundation, Switzerland.

The fractures were scored according to the Müller AO Classification of Fractures of Long Bones [18]. The intra-rater reliability for this scoring was acceptable (Kappa = 0.510). According to this classification system, the fractures are divided into A (extra-articular), B (partial intra-articular), and C (complete intra-articular) fractures. A, B, and C fractures are each divided into three subgroups as shown in Figure 1.

Intra-articular fractures (B and C) which we considered as more severe were compared with extra-articular fractures (A) [19].

Furthermore, other possible radiological predictors of functional outcome including dorsal angel, carpal malalignment, articular step, dorsal communition, and ulnar variance were noted [5,19]. Ulnar variance was defined as the difference in length of the ulna and radius at the injured wrist compared to the normal uninjured side. As it was not routine to take radiographs of the uninjured hand at the hospital, only 54 patients had radiographs also from the uninjured hand. We defined >10° dorsal angulation and >3 mm ulnar variance as a more severe fracture.

### Statistical analyses

Continuous variables were presented as mean with standard deviation and categorical variables as numbers and percentages. Normality was checked by visual inspection of qq plot. For group comparison, we used *t* test or ANOVA for continuous variables and chi-square test for categorical variables. For non-normally distributed parameters, Mann Whitney *U* or Kruskal-Wallis was used.

The clinical characteristics in Table 1 were chosen for their potential influence on fracture both independently of BMD and via BMD. The association between the extra- and intra-articular fractures as dependent variable and independent variables (Table 1) were tested separately in unadjusted logistic regression analyses and also in adjusted analysis one by one. The degree of association was expressed as odds ratio (OR) with 95% confidence intervals (CI). For these analyses, we used BMD values in mg/cm^2^.

**Table 1:** **Demographic variables, clinical characteristics, and bone density**

	**AO fracture group**			***P*** **values**	
	**A**	**B**	**C**	**All three fracture groups**	**Extra- (A) versus intra-articular (B, C) fracture**
	***n*** **= 83**	***n*** **= 5**	***n*** **= 22**		
Age (year)	68.2 (10)	69.9 (12.5)	69.8 (8.9)	0.763	0.449
Height (cm)	164.5 (5.8)	163.2 (9.4)	166 (5.9)	0.493	0.491
Weight (kg)	68.0 (13.3)	67.3 (13.1)	72.4 (11.7)	0.374	0.220
Smoking current	11/83	0/5	3/21^a^	0.674	0.560
Current exercise^b^	65/83	3/5	13/21	0.232	0.076
Chronic disease^c^	17/83	0/5	10/22	0.023	0.072
GC, current use	3/81^a^	0/5	2/19^a^	0.106	0.131
GC, ever use	5/81^a^	1/5	5/19^a^	0.028	0.016
Calcium, current use	12/80^a^	1/5	5/21^a^	0.662	0.341
Vitamin D, current use	12/80^a^	1/5	4/21^a^	0.877	0.406
Estrogen, current use	4/80	0	2/21	0.621	0.457
Bisphosphonate, current use	6/80^a^	1/5	2/21^a^	0.612	0.385
DXR BMD (g/cm^2^)	0.487 (0.079)	0.492 (0.109)	0.479 (0.053)	0.895	0.208
DXA fn BMD (g/cm^2^)	0.789 (0.130)	0.804 (0.770)	0.827 (0.130)	0.464	0.223
DXA tot hip BMD (g/cm^2^)	0.828 (0.119)	0.829 (0.132)	0.887 (0.116)	0.164	0.100
DXA spine BMD (g/cm^2^)	1.031 (0.174)	1.004 (0.168)	1.114 (0.242)	0.172	0.714

*GC* glucocorticoids, *BMD* bone mineral density, *DXR* digital X-ray radiogrammetry, *DXA* dual energy X-ray absorptiometry, *fn* femoral neck. ^a^The number differs due to missing values; ^b^exercise at least 30 min three times a week; ^c^inflammatory or endocrine chronic disease, RA 3, other rheumatic diseases 5, asthma 11, hypothyroidism 5, diabetes mellitus 5, hyperparathyroidism 1, other endocrine diseases 2. Distal radius fracture scored according to Müller AO Classification of Fractures of Long Bones. Continuous variables are presented as mean with standard deviation and categorical variables as numbers.

We also performed logistic regression analyses using the radiologic variables dorsal angle, carpal malalignment, articular step, and ulnar variance as dependent variables. Spearman correlation coefficient was used to explore the association between BMD and dorsal angle and ulnar variance.

Statistical tests were performed using PASW Statistics 18 (IBM SPSS statistics), and significance level was *p* < 0.05.

The study was approved by the Regional Ethical Committee (REK ‘HELSE SØR’, approval number S-03207).

## Results

Among the 218 women attending the osteoporosis center, no statistically significant differences for age (68.6 vs. 69.1 years = 0.77 CI difference −3.3 to 2.2), weight (68.8 vs. 68.9 kg, *p* = 0.65, CI difference −1.2 to 2.2), or height (164.7 vs. 164.2 cm, *p* = 0.86, CI difference −3.4 to 3.4) were found between women who had radiographs for DXR assessment (*n* = 110) available or not (*n* = 108).

Mean age (SD) among the 110 women with DXR-BMD was 68.6 (9.8) years (range 50–96). All women except of two (age 53 and 55 years) had been post-menopausal for more than 12 months. Among them, 83 patients had A fractures (0 A1, 34 A2, 49 A3), 5 had B fractures (4 B1, 1 B2), and 22 had C fractures (17 C1, 3 C2 and 2 C3). Hence, 75% had an extra-articular fracture (A2 or A3), and 25% had an intra-articular fracture (B or C).

In Table 1, demographic, clinical, and BMD data are displayed for patients in the fracture groups A, B, and C. A statistical significant difference between the fracture groups was found for patients having a chronic inflammatory or endocrine disease and for ever users of glucocorticoids (GC). Between patients with intra- and extra-articular fractures, a significant difference was only found for ever users of GC.

### The association between distal radius fracture severity and potential risk factors tested in unadjusted and adjusted models

In unadjusted logistic regression, DXR-BMD was not significantly associated with intra/extra articular fracture (Table 2). Adjusting for possible confounders from Table 1, one by one did not change the result for DXR-BMD considerably (data not shown). For the other BMD measures, we did the same analysis adjusting for possible confounders with the same result; neither of them was associated with fracture severity (data not shown). As shown in Table 2, only ever use of GC was significantly associated with having an intra-articular fracture in unadjusted analysis. Ever use of GC remained significantly associated with an increased risk of having an intra-articular distal radius fracture in the analysis with BMD measures and also when adjusted for age, chronic disease, and factors from Table 1 that might have had a potential influence on the use of GC (GC adjusted OR 4.2, *p* = 0.04).

**Table 2:** **Potential associates with intra-articular distal radius fracture assessed in unadjusted logistic regression analyses**

	**OR (95% CI)**	***P*** **value**
Age (years)	1.017 (0.973–1.063)	0.457
Height (cm)	1.029 (0.955–1.108)	0.459
Weight (kg)	1.020 (0.987–1.054)	0.245
Current smoking	0.854 (0.219–3.327)	0.820
Current exercise^a^	0.443 (0.172–1.142)	0.092
Chronic disease^b^	2.284 (0.887–5.880)	0.087
Ever use of GC	5.067 (1.390–18.466)	0.014
Current use of GC	3.754 (0.698–19.756)	0.124
Current use of calcium	1.700 (0.566–5.105)	0.344
Current use of vitamin D	1.349 (0.426–4.271)	0.610
Current use of estrogen	1.583 (0.273–9.188)	0.608
Current use of bisphosphonates	1.609(0.373–6.946)	0.524
DXR hand BMD (mg/cm^2^)	0.999 (0.993–1.005)	0.737
DXA femoral neck BMD (mg**/**cm^2^)	1.002 (0.999–1.006)	0.235
DXA total hip BMD (mg/cm^2)^	1.003(0.999–1.007)	0.103
DXA spine BMD (mg/cm^2^)	1.002 (0.999–1.004)	0.143

*OR* odds ratio, *CI* confidence intervals, *GC* glucocorticoids, *DXR* digital X-ray radiogrammetry; *BMD* bone mineral density, *DXA* dual energy X-ray absorptiometry. ^a^Exercise at least 30 min three times per week; ^b^inflammatory or endocrine chronic disease, rheumatoid arthritis 3, other rheumatic diseases 5, asthma 11, hypothyroidism 5, diabetes mellitus 5, hyperparathyroidism 1, other endocrine diseases 2.

### Radiographic fracture severity defined as dorsal angle, carpal malalignment, articular step, dorsal communition, and ulnar variance

A statistical significant correlation was found between DXR-BMD and the dorsal angle (spearman *r* = −0.219, *p* = 0.026) and ulnar variance (*n* = 54, *r* = −0.273, *p* = 0.045) at fracture time; however, no significant correlation was found with DXA at the spine L2–4 (*r* = −0.065, *p* = 0.52; *r* = 0.085, *p* = 0.54), femoral neck (*r* = −0.121, *p* = 0.23; *r* = −0.180, *p* = 0.20), or total hip (*r* = −0.215, *p* = 0.12; *r* = −0.199, *p* = 0.15), respectively. When dorsal angle with cutoff 10° and ulnar variance with cutoff 3 mm as dependent variables were tested in logistic regression analysis, none of the variables listed in Table 1 were significantly associated with the defined fracture severity outcomes.

For articular step in C fractures, carpal malalignment, and dorsal communition, no significant differences were observed for DXR-BMD or the other variables in Table 1.

## Discussion

We did not observe any significant association between decreased hand cortical DXR-BMD and the risk of having an intra-articular or extra-articular fragility fracture in distal radius scored according to the AO classification. A small significant negative correlation was found between lower hand DXR-BMD and initial fracture displacement measures as dorsal angle and ulnar variance; however, no significant association was found with carpal malalignment, articular step, or dorsal communition of the distal radius fracture. We did not find any association between the BMD at hip and spine measured by DXA and fracture severity.

Previous *in vivo* studies have reported inconsistent findings on the association between DXA-BMD and fracture severity. Some have reported a weak association whereas others have reported no significant association between fracture severity and BMD [7-11,20]. Clayton et al. found that DXA-BMD at the hip was lower in patients with an extra-articular fracture than that in patients with an intra-articular fracture [7]. On the other hand, Hollevoet et al. using DXA-BMD of the non-injured forearm found no significant difference in DXA-BMD between patients with intra-articular versus extra-articular fractures but reported that increased ulnar variance correlated inversely with DXA-BMD [10]. Xie and Barenholdt also found no difference in DXA spine or femoral neck BMD between patients with a displaced distal radius fracture and an undisplaced fracture [11]. However, for peripheral QCT measures, they found a lower cortical but not a lower trabecular bone density in patients with a displaced fracture compared with undisplaced fractures [11]. Our results are in line with previous reports showing no association between DXA-BMD and distal radius fracture severity [10,11]. In our study, however, a weak association was found between cortical hand BMD measured by DXR and some radiographic measures of fracture severity (dorsal angle and ulnar variance). No association was, however, observed between cortical hand BMD and the AO classification of intra- and extra-articular fracture or the articular step or dorsal communition of the distal radius fracture. This indicates that neither central BMD nor peripheral cortical BMD is a major determinant of the severity of distal radius fracture. This is in contrast to the reported association between low BMD and distal radius fracture risk, where both centrally and peripherally reduced BMD, including DXR-BMD measurements, have been shown to be significant and consistent risk factors for fragility distal radius fracture [2,15]. Other factors than BMD, like bone quality parameters and fall pattern, may therefore more likely be responsible for fracture type and fracture severity. In a hip fracture study, no association between bone mineral density measured with DXA and the severity of fracture was found [21]. In a study exploring fracture at proximal humerus using MicroCT, no association between local bone structure and cortical index and severity of fractures was found, which led the authors to conclude that a complex fracture do not necessarily imply lower bone quality compared to simple fracture [22].

Interestingly, our data indicate that GC use may be associated with the occurrence of intra-articular distal radius fracture. GC is known both to reduce BMD and impair bone quality and has been shown to increase fracture risk independently of BMD [23,24]. This has also been reported for distal radius fracture [25]. Other factors have also been linked to fracture severity. In the hip fracture study by Larrosa et al., the authors concluded that a more severe vitamin D deficiency seems to be associated to more severe osteoporotic hip fractures [26]. These observations add evidence to the hypothesis that distal radius fracture severity is more associated with other factors compromising bone quality than BMD. There is an increased interest in studying other bone features than BMD contributing to bone strength. Techniques such as high-resolution quantitative computed tomography (HR-QCT), high-resolution magnetic resonance imaging (HR-MRI), and quantitative ultrasound (QUS) can be used for evaluation of bone quality *in vivo* where QUS has the advantage of being none ionizing and also carry information about tissue properties beyond microstructure [26,27]. These features might give better understanding of bone properties and their implication on the severity of fracture.

Our study has limitations that should be considered when interpreting the findings. The number of studied fracture patients is only 110. However, compared with previous studies, the number of patients is in the upper range. In the previous studies, the number of included women with fragility distal radius fracture ranged from 40–127 patients [7-10,20]. Another limitation is that for DXA spine, we used L2–L4 and not the L1–L4 measurement site which later has been recommended as measurement site for the spine by the International Society for Clinical Densitometry (ISCD). This may have influenced the results. However, studies have shown that precision expressed by CV% for Lunar spine DXA is similar for L2–L4 and the L1–L4 measurements [28]. The X-rays were analyzed retrospectively, and we therefore relied on the decision of the treating surgeons concerning what X-ray had been taken. For the first patients included, some X-rays were scanned from conventional X-rays leading to possible dispersion in the radiographic measures. Further, better intra-reader variability for the AO classification may have improved the results in our study. This variability was, however, at the same level as reported by others [29]. Ideally, all X-rays for the DXR measurement should have been performed on the non-dominant hand; however, this was not always possible when the fracture was in non-dominant hand because of technical difficulties in positioning the hand (17 cases). There is no gold standard defining fracture severity in the orthopedic surgeon community. Several fracture classification systems and variables have been developed and used to describe the fracture type and severity. The measures we are reporting are thought to be of clinical relevance for treatment decision and as prognostic factors for radiographic and clinical outcome [19].

Information about, e.g., diseases and medical treatments was self-reported. Retrospective data collection can be biased by recall problems and response shifts due to fracture. Given the relative short time between fracture and data collection (median 10 days), we believe that this is a minor problem in our study. Ideally, we should have had information about the doses of GC which was used in the individual patients. However, from epidemiological studies, we know that even prednisolone doses as low as 2.5 mg daily increase fracture risk [30].

## Conclusion

BMD neither measured centrally at the spine or hip nor locally in the hand seems to correlate substantially with the type or severity of fragility fracture in the distal forearm in women. Only a weak association was found between cortical hand DXR-BMD and some radiographic measures used to describe fracture severity including ulnar variance and dorsal angle. The use of GC seems to increase the risk of having an intra-articular fracture, suggesting that bone material properties more than bone density may impact on the risk of having a more severe fracture.

## Abbreviations

BMD: bone mineral density
CI: confidence Intervals
DXA: dual energy X-ray absorptiometry
DXR: digital X-ray radiogrammetry
fn: femoral neck
GC: glucocorticoid
RA: rheumatoid arthritis
OR: odds ratio
